# Spinal Cord Glycine Transporter 2 Mediates Bilateral ST35 Acupoints Sensitization in Rats with Knee Osteoarthritis

**DOI:** 10.1155/2019/7493286

**Published:** 2019-02-07

**Authors:** Fuhai Bai, Yongyuan Ma, Haiyun Guo, Yuheng Li, Feifei Xu, Ming Zhang, Hailong Dong, Jiao Deng, Lize Xiong

**Affiliations:** Department of Anesthesiology and Perioperative Medicine, Xijing Hospital, the Fourth Military Medical University, Xi'an 710032, Shaanxi, China

## Abstract

The concept of “acupoint sensitization” refers to the functional status of acupoint switches from silent to active under pathological conditions. In clinic, acupoint sensitization provides important guidance for acupoints selection in different diseases. However, the mechanism behind this phenomenon remains unclear. We generated a model of knee osteoarthritis (KOA) by intra-articular injection of monosodium iodoacetate (MIA) into the left knee of rats. The paw withdrawal mechanical threshold (PWMT) and the total number of mast cells as well as mast cell degranulation rate (MCDR) of acupoint tissue were used to test whether the acupoints were sensitized. The results showed that KOA resulted in a reduced mechanical threshold and elevated total number of mast cell as well as mast cell degranulation rate at bilateral ST35 (Dubi) but not GB37 (Guangming) or nonacupoint area. The acupoint sensitization was accompanied by upregulation of glycine transporter 2 (GlyT2) and reduction of extracellular glycine levels in the bilateral dorsal horns of the spinal cord at L3-5. Selective inhibition of GlyT2 or intrathecal administration of glycine attenuated ST35 acupoint sensitization. The sensitization of bilateral ST35 was blocked after intraspinal GlyT2 short hairpin (sh) RNA (GlyT2-shRNA) microinjection to specifically downregulate GlyT2 expression in the left side (ipsilateral) L3-5 spinal cord dorsal horn before MIA injection. Moreover, electroacupuncture (EA) stimulation at ST35 ameliorated articular pathological lesions and improved KOA-related pain behaviors. GlyT2-shRNA injection reversed EA-induced pain relief but not EA-induced reduction of joint lesions. Overall, this study demonstrated that spinal GlyT2, especially elevated GlyT2 expression in the ipsilateral dorsal horn of the spinal cord, is a crucial mediator of ST35 acupoint sensitization in KOA rats.

## 1. Introduction

Acupoints are special sites on the body surface or under the skin along the meridians. The functional status of certain acupoints switches from silent to active when the body is under pathological conditions. This phenomenon is called “acupoint sensitization” [1, 2]. Specifically, when disease strikes, responsive acupoints will appear to have increased sensitivity to pressure, heat, light, or electric stimuli, and the therapeutic effects of acupuncture or moxibustion at sensitized acupoints will be enhanced. Acupoint sensitization provides important guidance for acupoints selection in clinical practice. However, the mechanism behind this phenomenon remains unclear.

Given that acupoint sensitization is mainly manifested through sensory changes, the nervous system is presumed to play a major part in this phenomenon. It has been reported that noxious visceral stimuli intensify the functional responses to stimulation at acupoints [3]. The discharge frequency of neurons in the ventral posterior lateral (VPL) nucleus was increased when stimulation at the “Zusanli-Shangjuxu” acupoints was applied to rats with colorectal distension compared to normal rats [3]. This result indicates that central nervous system (CNS) sensitization may be involved in acupoint sensitization. Glycine is an important inhibitory neurotransmitter in the spinal cord. Reduction of spinal glycinergic neurotransmitters may contribute to central sensitization [4]. Since the concentration of glycine in the synaptic cleft is modulated by glycine transporter 1/2 (GlyT1 and GlyT2) [5], alteration of their function or expression may have significant effects on acupoint sensitization. Therefore, the aim of this study was to reveal whether changes in the spinal cord dorsal horn, especially the glycinergic system, are involved in the initiation of acupoint sensitization in a rat model of KOA.

KOA is a type of joint diseases that results from breakdown of knee joint cartilage and underlying bone. Globally, as of 2010, approximately 250 million people had osteoarthritis of the knee [6, 7]. The prevalence, disability, and associated costs of KOA are expected to continue growing over the next 25 years because of the aging of society [8, 9]. Acupuncture has been widely verified to be effective in slowing the progression and relieving pain for KOA patients by randomized controlled trials [10, 11] and systematic reviews [12–14]. The phenomenon of acupoint sensitization has been described in a rat model of KOA [15]. The current study employed KOA as disease model to explore whether GlyT1/2 is involved in the development of acupoint sensitization during KOA, providing new insights into the mechanism of peripheric acupoint sensitization.

## 2. Materials and Methods

### 2.1. Animals and Left Side KOA Model

Male Sprague-Dawley rats weighing 200-250 g were obtained from Beijing Vital River Laboratory Animal Technology Co., Ltd., Beijing, China. Rats were housed in standard breeding cages and maintained on a 12-h light/dark cycle at 21 ± 2°C with food and water* ad libitum*. All animal procedures were approved by the Ethics Committee for Animal Experimentation of the Fourth Military Medical University (Xi'an, China) and in accordance with the National Institutes of Health Guide for the Care and Use of Laboratory Animals.

The unilateral KOA rat model was described in more details in our previous report [16]. Briefly, all surgical procedures were performed under anesthesia with 1.5% isoflurane in oxygen. One milligram of MIA (Sigma-Aldrich, USA) was dissolved in 50 *μ*L sterile saline [17]. In the left knee, a single intra-articular injection of 1 mg MIA was performed by inserting a 31-gauge needle through the infrapatellar ligament into the joint space. Animals in the control group received 50 *μ*L saline instead.

### 2.2. Behavioral Tests

PWMT test: Mechanical hyperalgesia was determined by measuring the incidence of foot withdrawal in response to mechanical stimulation of the plantar surface of left side (ipsilateral) hind paw using a sharp, cylindrical probe with a uniform tip diameter of approximately 0.2 mm (ALMEMO 2390-5 Electronic von Frey Anesthesiometer; IITC Life Science, USA). Each rat was tested six times, and the mean value was defined as the PWMT of the tested subject [18].

Measurement of weight-bearing deficit: Briefly, changes in weight bearing were measured using a weight in capacitance tester (IITC Incapacitance Meter, USA) as previously described [16]. We documented five measurements of the weight borne on each hind paw. The percentage of ipsilateral weight bearing was calculated as [(weight borne on ipsilateral paw/sum of the weight borne on the ipsilateral and contralateral paws) × 100%]. The mean value of the 5 tests was obtained as the weight born of the tested rat.

**Figure 1:**
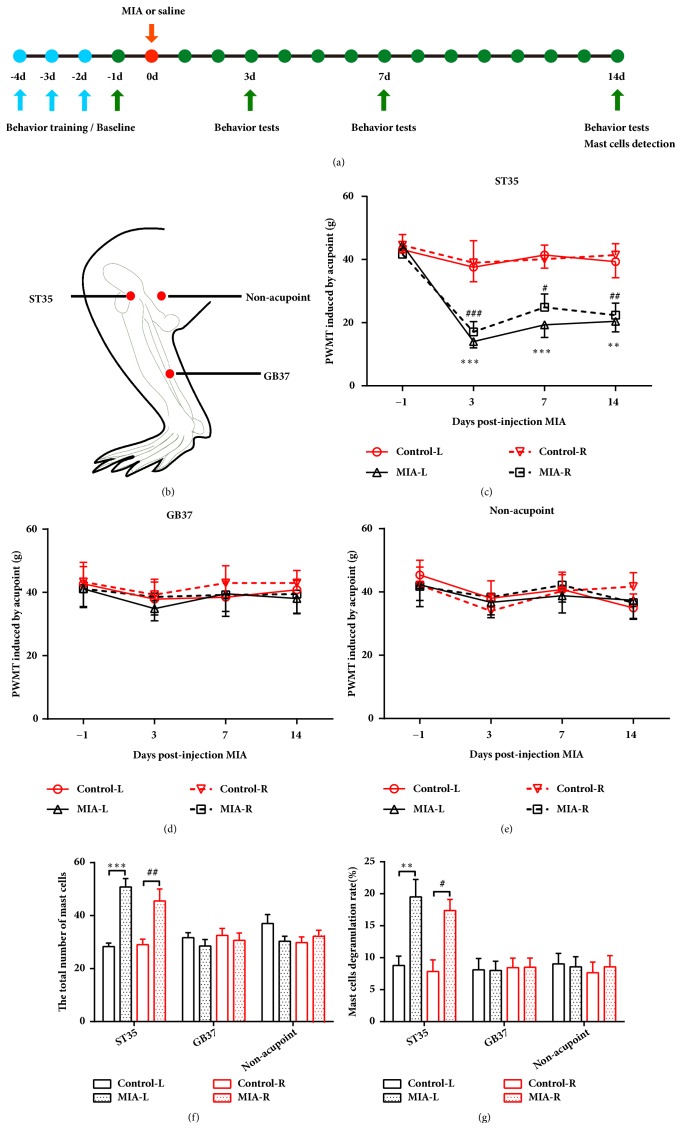
Left KOA induces bilateral ST35, but not GB37 or nonacupoint control area sensitization. (a) Schematic diagram for the time frame of the experiment. (b) The locations of ST35, GB37, and the nonacupoint area. (c-e) Paw withdrawal mechanical threshold at ST35, GB37, and the nonacupoint area. Two-way repeated measures ANOVA followed by Bonferroni's post hoc test was used, *∗∗P* < 0.01, and *∗∗∗P* < 0.001 versus Control-L group; ^#^*P* < 0.05, ^##^*P* < 0.01, and ^###^*P* < 0.001 versus Control-R group; n = 8 per group. ((f) and (g)) The total number of mast cells and the percentages of degranulated mast cells in all groups. One-way ANOVA test followed by Tukey's post hoc test was used, *∗∗P* < 0.01, and *∗∗∗P* < 0.001 versus Control-L group; ^#^*P* < 0.05,^ ##^*P* < 0.01 versus Control-R group; n = 6 per group. All data are shown as the mean ± SEM.

Acupoint sensitization tests: A sharp, cylindrical probe with a uniform tip diameter of approximately 0.2 mm (ALMEMO 2390-5 Electronic von Frey Anesthesiometer; IITC Life Science, USA) was used. Acupoints mechanical threshold were determined by measuring the incidence of foot withdrawal in response to mechanical indentation of the ST35, nonsensitized acupoints GB37, and a nonacupoint area (located 5 mm behind the ST35) on bilateral hind limbs. The acupoints chosen for behavioral testing were based on traditional Chinese medicine meridian theory [16, 19]. The probe was separately applied to 6 designated acupoints distributed over the bilateral hind limbs surface (Figure 1(b)). The minimal force (in grams) that induced paw withdrawal was read off the display. The mechanical threshold of acupoint was calculated as the mean of the 6 readings. All behavior tests were examined by two independent and blinded investigators.

### 2.3. EA Treatment

EA treatment was performed as previously described in our report [16]. Awake rats were restrained gently in an immobilization apparatus with their hind limbs exposed and hind paws on the floor. The rats were acclimatized in this apparatus for 30 min per day on 3 consecutive days before tests. Two acupuncture needles were inserted into the bilateral ST35 and stimulated with 1 mA, 2/15 Hz electric current for 30 min (SDZ-V Huatuo Electroacupuncture Instrument, Suzhou Medical Appliances Co., Ltd., Suzhou, China). For sham EA, the needles were inserted superficially into the subcutaneous layer at nonacupoints (localized 5 mm behind the ST35) without electrical stimulation. EA or sham EA was given once per day for 6 consecutive days followed by a 1-day break for 2 weeks.

### 2.4. Immunohistochemical Staining

Rats were decapitated under deep anesthesia after the last behavioral test and at 14 days after MIA injection, and skin samples of 1.5×1.5×1.5 mm^3^ at the ST35, GB37, and nonacupoint control area [15] were collected and fixed in 4% paraformaldehyde (PFA). The tissues were then dehydrated, embedded in paraffin, and sectioned into 5 *μ*m thick slices. Subsequently, the sections were dewaxed, dehydrated, and stained in 0.5% toluidine blue for 30 min and then washed with tap water. After vitrification with dimethylbenzene and neutral balata fixation, the sections were observed under a light microscope (BX51, Olympus Corporation, Japan). Mast cell degranulation was defined as >10% of the granules exhibiting fusion or discharge and, for each acupoint, the total number of mast cells and degranulated mast cells were counted in five separate fields under 40 × magnification and the sum was used to represent the number per animal. Samples from 6 animals were used [15, 20]. The percentage of degranulated mast cells was calculated as [(degranulated mast cells/sum of the number of mast cells) × 100%]. The results were expressed as the total number of mast cells and the percentage of mast cell degranulation.

### 2.5. Joint Pathology

Rats were decapitated under deep anesthesia, and left knee joints were dissected and fixed with 4% PFA for 24 h. The explanted joints were decalcified in formic acid (5%) with 20% EDTA for 3 weeks before incubation with ammonia solution for 30 min to ensure neutralization and rinsed in water for 24 h. After that, the joints were dehydrated using an ascending ethanol series and embedded in paraffin. The samples were coronally sectioned to a thickness of 5 *μ*m and stained with hematoxylin and eosin (H&E). According to the Osteoarthritis Research Society International (OARSI) 0-24 point score system, the progression depth of osteoarthritis into cartilage and the completeness of the articular surface were evaluated by two independent investigators [21]. All sections were counted by an experimenter blinded to the treatment groups.

### 2.6. Jugular Catheter and Drug Administration

Rats were anesthetized and placed in a supine position, and the skin of the right ventral side of the neck and clavicle area were shaved and sterilized. A 1-2 cm incision was made lateral to the trachea, and the jugular vein was exposed and dissected. A 4-0 silk suture was placed posterior to the vein with a PE10 catheter inserted into the superior vena cava and the suture tightened around it to stop bleeding. Then, the incision was closed with 3-0 monofilament suture [22]. The other side of the catheter was tunneled under the skin to the dorsal cervical region, flushed with saline, and sealed with a cautery pen. One mg/kg GlyT2 inhibitor, ORG25543, was intravenously delivered through the catheter once every 48 h after MIA injection for KOA model establishment. Drugs were administered a total of 7 times during the 14-day period [23–25].

### 2.7. Intrathecal Catheter Implantation and Glycine Infusion

Rats were anesthetized and placed on a stereotactic frame in the prone position. The skin was shaved and sterilized, and a midline skin incision (2-3 cm) was made at the lumbar enlargement. The superficial muscle was dissected from the spinous process. A saline-filled PE-10 was inserted into the subarachnoid space between L5 and L6, embedded through a tunnel under the skin through up to the back of the neck, and connected with a microinjection pump (ALZET Miniosmotic pump, model 2002, USA). A minipump was implanted subcutaneously at the back of the neck and connected to the tube and pumping glycine or saline at a rate of 0.5 *μ*L/h for 14 days. Glycine was dissolved in saline (0.9%) at a concentration of 0.2 mol/L.

### 2.8. Spinal Cord Microdialysis

Rats were anesthetized, and the core body temperature was maintained at 37 ± 0.5°C using a homeothermic blanket (Xi'an Wandong Instrument Co., Ltd., China). Rats were placed on a stereotactic frame in the prone position. The skin of the dorsal aspect of the lumbar enlargement was shaved and sterilized. A midline incision was made, and muscle was separated from the spinous process. Then, the vertebra of L3 was held immobilized using a spinal clamp. A microdialysis probe was inserted into the spinal cord 0.6 mm lateral to the midline at an angle of 16° from the horizontal [26]. A dialysis membrane (TP-100-10, Eicom, Japan) of 1.5 mm length was inserted into the dorsal horn of the L3-L5 lumbar region of the spinal cord. The probe was perfused with an artificial cerebrospinal fluid (ACSF: 140.0 mM NaCl, 3.0 mM KCl, 1.5 mM CaCl_2_, 1.0 mM MgCl_2_, 1.5 mM Na_2_HPO_4_, 0.27 mM NaH_2_PO_4_, and pH 7.4). Dialysates in bilateral dorsal horns of spinal cord were collected at a rate of 2 *μ*L/min for 30 min after 120 min equilibration. The concentration of glycine was detected with fluorescence by high-performance liquid chromatography (HPLC) [26].

### 2.9. Western Blot Analysis

Rats were decapitated under deep anesthesia, and the lumbar enlargement of the spinal cord was removed. The dorsal horn region of the spinal cord was horizontally separated with a vibratome in ice-cold ACSF. Then, the left side (ipsilateral KOA) and right side (contralateral KOA) of the spinal cord dorsal horn were collected. Samples were immediately frozen in liquid nitrogen and stored at -80°C until use. Tissue was homogenized in RIPA buffer containing 1% protease cocktail inhibitor (Sigma, USA) on ice for 10 min and then centrifuged at 12000 rpm/min at 4°C for 20 min, and supernatants were collected. Protein concentration was determined using a bicinchoninic acid (BCA) protein assay kit (Sigma, USA). Thirty micrograms of protein samples was loaded for electrophoresis and then transferred onto a 0.45 *μ*m PVDF membrane (GE Healthcare). The membranes were blocked with 3% bovine serum albumin for 1 h followed by incubation with polyclonal rabbit anti-GlyT1 (1:200; Alomone labs, Israel), polyclonal rabbit anti-GlyT2 (1:1000; SYSY, Germany), polyclonal rabbit anti-c-Fos (1:1500; SYSY, Germany), or polyclonal rabbit anti-*β*-actin (1:1000; GeneTex, USA) primary antibodies overnight at 4°C. The membranes were rinsed 10 min × 3 times followed by incubation with goat-anti-rabbit antibody (1:4000; GeneTex, USA) secondary antibody for 1 h at room temperature and rinsed 10 min × 3 times before application of luminal reagent (Millipore, Germany) for visualization. Each sample was independently tested three times.

### 2.10. Immunofluorescent Staining

On the fourteenth day after MIA injection, rats were anesthetized and transcardially perfused with ice-cold saline (0.9%) followed by 4% PFA in 1 × phosphate buffered solution (PBS). The lumbar enlargement of the spinal cord was removed and fixed in 4% PFA for 24 h. Then, the samples were gradually dehydrated in a gradient sucrose of 20% and 30%. Thirty-micron-thick transverse sections were incubated with 10% normal goat serum for 1 h. After blocking, sections were probed with polyclonal guinea pig anti-GlyT2 (1:1500; SYSY, Germany) or polyclonal rabbit anti-c-Fos (1:1500; SYSY, Germany) antibodies in 1% bovine serum albumin-PBS overnight at 4°C in a humidified box. After washing in PBS for 10 min × 3 times, the secondary antibodies (594 goat anti-rabbit lgG, 1:500; 488 goat anti-guinea pig lgG, 1:500; Life Technologies, USA) were applied onto the slides for 1 h at 37°C. Finally, sections were observed under a fluorescence microscope (Olympus BX51, Japan).

### 2.11. Intraspinal Microinjection for Virus Administration

To specifically knockdown GlyT2 expression in the ipsilateral spinal cord, we constructed recombinant adeno-associated virus (AAV) containing full-length rat GlyT2 short hairpin (sh) RNA (GlyT2-shRNA) or a negative control-shRNA (Con-shRNA), which also carried an enhanced green fluorescent protein (EGFP) and was injected into the ipsilateral L3-L5 spinal cord dorsal horn (Shanghai Taitool Bioscience Co., Ltd., China). A laminectomy was performed to expose the dorsal horn of the L3-L5. The coordinate was 0.7 mm left to the midline at a depth of 0.4 mm [27]. One microliter of GlyT2-shRNA (*⩾*2 × 10^12^ vg/ml) was injected into two ipsilateral intervertebral spaces (L3/L4 and L4/L5) using a glass micropipette at a rate of 46 nL/min. In control group, 1 *μ*l of Con-shRNA was also injected into the same sites. Ten minutes after the injection was finished, and micropipettes were slowly retracted.

### 2.12. Statistical Analysis

Data are presented as the mean ± SEM for behavioral results and the mean ± SD for OARSI scores. Using GraphPad Prism 6.02 software, pain behaviors were analyzed with two-way ANOVA for repeated measurements, followed by Bonferroni's post hoc test. One-way ANOVA test followed by Tukey's post hoc test was used for mast cells, western blot, and immunofluorescent staining analysis when four groups were compared. Kruskal-Wallis test followed by post hoc Dunn's tests was used for OARSI score analysis in four group comparisons.* P* values less than 0.05 were considered statistically significant.

## 3. Results

### 3.1. Left Knee Osteoarthritis Induces Bilateral ST35 Acupoints Sensitization

**Figure 2:**
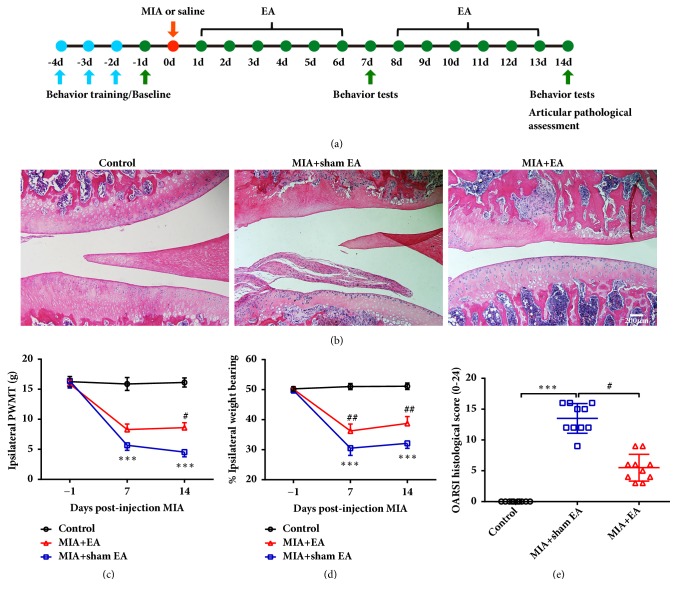
Acupuncture at bilateral ST35 acupoints improved KOA relative behaviors and articular pathology score. (a) Schematic diagram for the time frame of the experiment. (b) Representative H&E staining images for articular pathology assessment in the MIA- and EA-treated groups. The scale bar is 200 *μ*m. (e) OARSI scores are presented as the mean ± SD; Kruskal-Wallis test was used and *∗∗∗P* < 0.001 versus Control group; ^#^*P *< 0.05 versus MIA+sham EA group; n = 10 per group. ((c) and (d)) Paw withdrawal thresholds of the ipsilateral hind paws and weight-bearing deficits were assessed in MIA- and EA-treated rats. Two-way repeated measures ANOVA followed by Bonferroni's post hoc test was used, *∗∗∗P* < 0.001 versus Control group; ^#^*P* < 0.05 and ^##^*P* < 0.01 versus MIA+sham EA group; n = 8 per group. Data are presented as the mean ± SEM.

To determine whether KOA induces certain acupoint sensitization, PWMT, the total number of mast cells, and the MCDR at a related acupoint (ST35), an irrelevant acupoint (GB37) and a nonacupoint control were assessed (Figure 1(b)). Bilateral PWMTs of ST35 were significantly reduced at days 3, 7, and 14 after MIA intra-articular injection in the left side knee (Figure 1(c)). However, the PWMT of GB37 and the nonacupoint groups remained unchanged (Figures 1(d) and 1(e)). Compared to control rats, the total number of mast cells and the MCDR at bilateral ST35 were significantly increased in KOA rats. However, the total number of mast cells and MCDR at bilateral GB37 and nonacupoints were similar to that of control rats (Figures 1(f) and 1(g)). According to traditional Chinese medicine theory, acupuncture at sensitized acupoints can produce optimum therapeutic effects. Therefore, we applied EA or sham EA treatment at bilateral ST35 and assessed pain-related behaviors. The results showed that EA at the sensitized acupoint (ST35) increased the PWMT and alleviated the weight-bearing deficiency in the ipsilateral hind limb of rats (Figures 2(c) and 2(d)). Moreover, EA improved pathological changes in the ipsilateral knee and decreased the OARSI score after 14 days of treatment (Figures 2(b) and 2(e)).

### 3.2. Left Knee Osteoarthritis Increases c-Fos Expression in Bilateral L3-5 Spinal Cord Dorsal Horns

**Figure 3:**
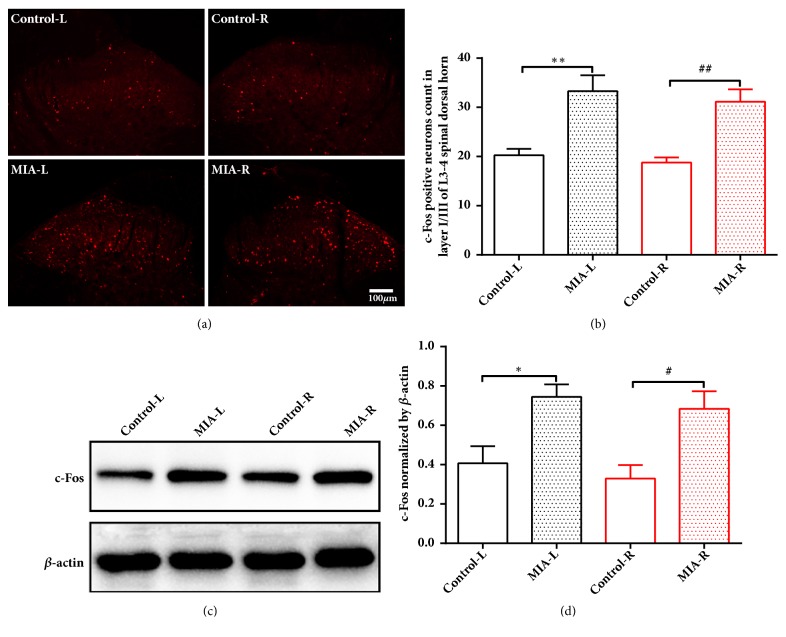
Left KOA induces c-Fos upregulation in bilateral dorsal horns of spianal cord. (a) Representative fluorescent staining image of c-Fos expression at L3-5 dorsal horn (red fluorescence) in control or MIA rats at 14 days after saline or MIA injection. (b) c-Fos-positive cell counts in L3-5, laminae I-III. Scale bar is 100 *μ*m; *∗∗P *< 0.01 versus Control-L group; ^##^*P* < 0.01 versus Control-R group; n = 8 per group. ((c) and (d)) Western blot analysis for the expression of c-Fos in bilateral L3-L5 spinal cord dorsal horn at 14 days after MIA or saline injection. *∗P *< 0.05 versus Control-L group; ^#^*P* < 0.05 versus Control-R group; n = 6 per group. One-way ANOVA followed by Tukey's post hoc test was used. All data are presented as the mean ± SEM.

Spinal sensitization was determined by measuring c-Fos expression in laminae I-III of the bilateral spinal cord dorsal horns. Immunofluorescent staining showed that KOA induced a robust increase in c-Fos expression in laminae I-III of bilateral L3-5 spinal cord dorsal horns at 14 days after MIA injection (Figures 3(a) and 3(b)). Consistently, Western blot revealed that the c-Fos protein level in bilateral spinal cord dorsal horns was significantly increased in KOA animals compared to control rats (Figures 3(c) and 3(d)).

### 3.3. Extracellular Glycine Concentration Reduction Contributes to Acupoint Sensitization

**Figure 4:**
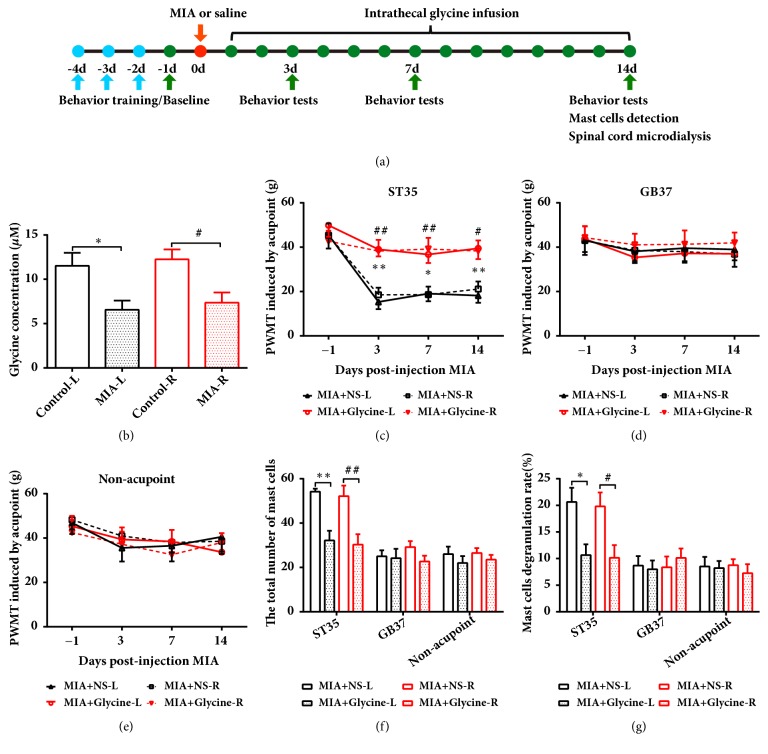
Intrathecal glycine administration attenuated acupoint sensitization in KOA rats. (a) Schematic diagram for the time frame of the experiment. (b) The concentration of glycine in bilateral spinal dorsal horn detected by microdialysis in KOA or control rats. One-way ANOVA followed by Tukey's post hoc test was used. *∗P* < 0.05 versus Control-L group; ^#^*P* < 0.05 versus Control-R group; n = 6 per group. (c-e) Paw withdrawal mechanical threshold in bilateral acupoints or nonacupoint areas of KOA and control rats. Two-way repeated-measures ANOVA followed by Bonferroni's post hoc test was used. *∗P *< 0.05 and *∗∗P* < 0.01 versus MIA+NS-L group; ^#^*P *< 0.05 and ^##^*P* < 0.01 versus MIA+NS-R group; n = 8 per group. ((f) and (g)) The total number of mast cells and the percentages of degranulated mast cells in all groups; n = 6 per group. One-way ANOVA followed by Tukey's post hoc test was used. *∗P *< 0.05 and *∗∗P* < 0.01 versus MIA+NS-L group; ^#^*P *< 0.05 and ^##^*P* < 0.01 versus MIA+NS-R group; n = 6 per group. All data are presented as the mean ± SEM.

To investigate whether glycine concentration in the extracellular area was changed after MIA injection, we used microdialysis to analyze the concentration of glycine in the extracellular dialysates on each side of the L3-L5 spinal dorsal horn. The results showed that the extracellular glycine concentration was decreased in the bilateral spinal cord dorsal horns at 14 days after MIA injection (Figure 4(b)), suggesting that the extracellular glycine content may play an important role in central sensitization.

Next, we sought to investigate whether acupoint sensitization was induced by extracellular glycine reduction in the spinal cord. Using a minipump, we infused glycine intrathecally at a speed of 0.1 *μ*mol/h in the spinal lumbar enlargement for 14 days, and the PWMTs of bilateral ST35 acupoints were significantly higher than that in saline infusion animals. However, the mechanical thresholds of GB37 and nonacupoint did not change after glycine infusion (Figures 4(c)–4(e)). The total number of mast cells and the MCDR of bilateral ST35 acupoints were significantly decreased after glycine infusion. However, no such variation was observed on either side of GB37 or nonacupoint area in saline-infused rats (Figures 4(f) and 4(g)). These results demonstrated that increased extracellular glycine could reverse the sensitization of bilateral ST35 without affecting the PWMT and the total number of mast cells or MCDR of nonsensitized acupoint.

### 3.4. GlyT2 but Not GlyT1 May Be Involved in the ST35 Sensitization in the Left KOA Rats

**Figure 5:**
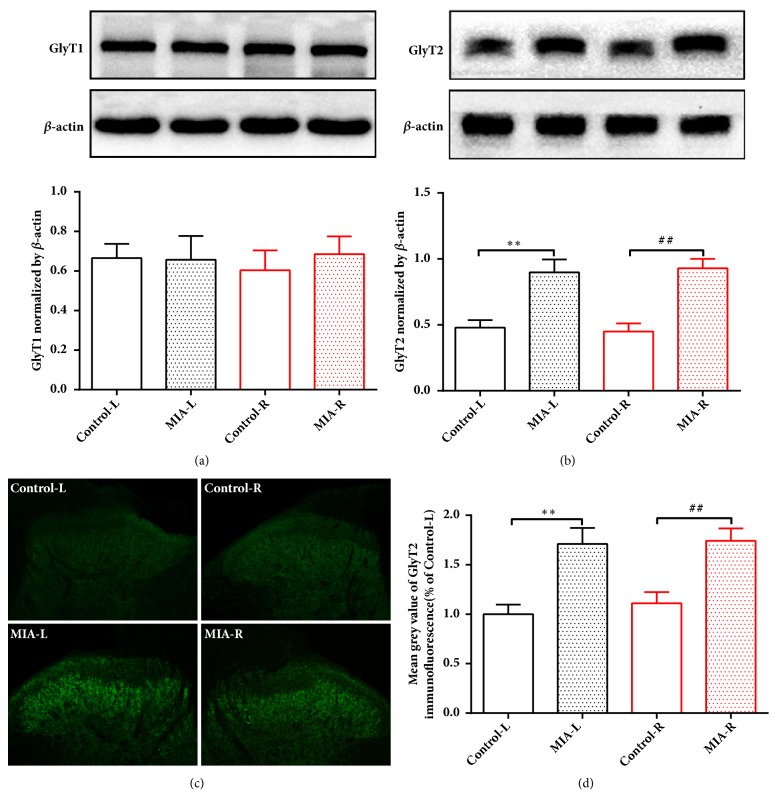
GlyT2 but not GlyT1 was increased in the left KOA rats. ((a) and (b)) Expression of GlyT1 and GlyT2 in bilateral L3-L5 spinal cord dorsal horn at 14 days after MIA or saline injection. *∗∗P* < 0.01 versus Control-L group; ^##^*P* < 0.01 versus Control-R group; n = 6 per group. (c) Representative fluorescent staining images (green fluorescence) of GlyT2 expression in the spinal cord dorsal horn at L3-5 in control and MIA rats. (d) Mean gray value of GlyT2 expression in L3-L5, laminae I-III. Scale bar is 100 *μ*m; *∗∗P* < 0.01 versus Control-L group; ^##^*P* < 0.01 versus Control-R group; n = 8 per group. All data are presented as the mean ± SEM; one-way ANOVA followed by Tukey's post hoc test was used.

Considering that glycine clearance and reuptake were carried out by glycine transporters (GlyT1/2), we next examined the expression of GlyT1/2 in the bilateral dorsal horns of spinal cord at 14 days after MIA injection. The results showed that the expression of GlyT1 was not changed in the bilateral spinal cord dorsal horns in KOA compared to control group (Figure 5(a)). However, GlyT2 was significantly increased in the bilateral spinal cord dorsal horns in rats from the KOA group (Figure 5(b)). This observation was further confirmed by fluorescent staining of GlyT2 expression (Figures 5(c) and 5(d)). Taken together, these results indicated that GlyT2, but not GlyT1, may be involved in the formation of central sensitization in KOA rats.

### 3.5. ST35 Sensitization Is Attenuated by ORG25543, a Selective Inhibitor of GlyT2

**Figure 6:**
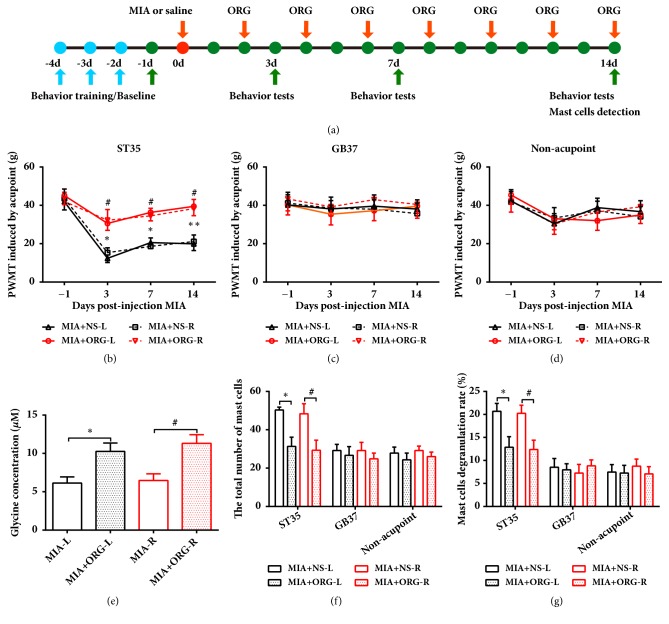
Selective inhibition of GlyT2 attenuated ST35 acupoint sensitization in KOA rats. (a) Schematic diagram for the time frame of the experiment. (b-d) Paw withdrawal mechanical threshold was assessed by stimulating at bilateral ST35, GB37, or nonacupoint. *∗P* < 0.05 and *∗∗P *< 0.01 versus MIA+NS-L group; ^#^*P* < 0.05 versus MIA+NS-R group; n = 8 per group. Two-way repeated-measures ANOVA followed by Bonferroni's post hoc test was used. (e) The concentration of glycine in bilateral spinal dorsal horn detected by microdialysis in unilateral KOA or ORG injected rats. One-way ANOVA followed by Tukey's post hoc test was used. *∗P* < 0.05 versus MIA+ORG-L group; ^#^*P* < 0.05 versus MIA+ORG-R group; n = 6 per group. ((f) and (g)) The total number of mast cells and the percentages of degranulated mast cells in all groups; One-way ANOVA followed by Tukey's post hoc test was used. *∗P* < 0.05 versus MIA+NS-L group; ^#^*P* < 0.05 versus MIA+NS-R group; n = 6 per group. All data are presented as the mean ± SEM. ORG: ORG25543.

GlyT2 is crucial for glycine reuptake into the presynaptic cytosol for refilling synaptic vesicles. Increased GlyT2 expression may lead to increased uptake of glycine from the synaptic cleft. Our results showed that intermittent intravenous injection of a GlyT2 selective inhibitor, ORG25543, significantly reversed the PWMT reduction of bilateral ST35 acupoints in KOA rats but did not affect the PWMT in GB37 or nonacupoint groups (Figures 6(b)–6(d)). Meanwhile, ORG25543 injection reduced the total number of mast cells and the MCDR at bilateral ST35, but not at bilateral GB37 or nonacupoint areas (Figures 6(f) and 6(g)) in KOA rats. The bilateral extracellular glycine concentrations were also increased after ORG25543 infusion in KOA rats (Figure 6(e)).

### 3.6. Downregulation of GlyT2 at the Spinal Cord Dorsal Horn Suppresses Acupoint Sensitization

**Figure 7:**
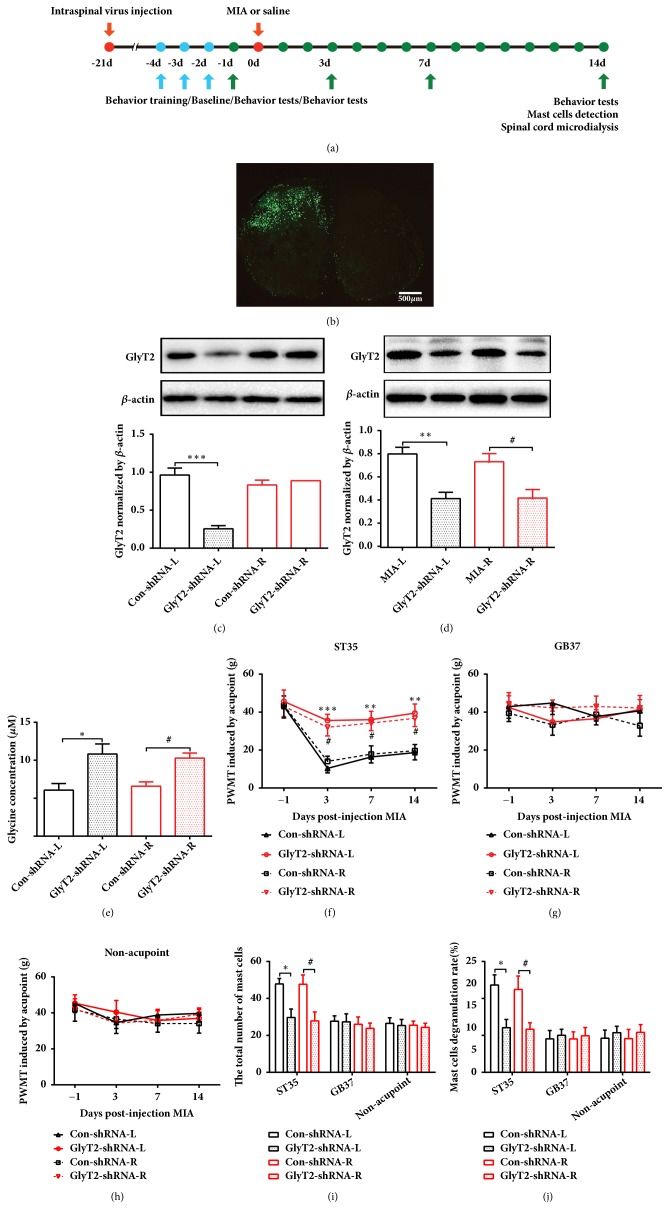
Ipsilateral downregulation of GlyT2 by GlyT2-shRNA attenuated ST35 acupoint sensitization in KOA rats. (a) Schematic diagram for the time frame of the experiment. (b) Representative image of GlyT2-shRNA expression in the L3-L5 ipsilateral spinal cord dorsal horn. The scale bar is 500 *μ*m. ((c) and (d)) Western blot analysis for the expression of GlyT2 at 21 days after GlyT2-shRNA injection in wild-type rats and unilateral KOA rats, respectively. One-way ANOVA followed by Tukey's post hoc test was used. *∗∗∗P* < 0.001 versus Control-shRNA-L group; n = 4 per group. (d) Western blot analysis for the expression of GlyT2 at 21 days after GlyT2-shRNA injection in unilateral KOA rats. One-way ANOVA followed by Tukey's post hoc test was used. *∗∗∗P* < 0.001 versus Control-shRNA-L group; n = 4 per group. (e) The concentration of glycine in the spinal dorsal horn detected by microdialysis after GlyT2-shRNA injection. One-way ANOVA followed by Tukey's post hoc test was used. *∗P* < 0.05 versus Control-shRNA-L group; ^#^*P *< 0.05 versus Control-shRNA-R group; n = 6 per group. (f-h) Paw withdrawal mechanical threshold at bilateral ST35, GB37 and nonacupoint area, respectively. Two-way repeated-measures ANOVA followed by Bonferroni's post hoc test was used. *∗∗P* < 0.01, *∗∗∗P* < 0.001 versus Control-shRNA -L group; ^#^*P* < 0.05 versus Control-shRNA-R group; n = 8 per group. ((i) and (j)) The total number of mast cells and the percentages of degranulated mast cells in all groups. One-way ANOVA followed by Tukey's post hoc test was used. *∗P* < 0.05 versus MIA+NS-L group; ^#^*P* < 0.05 versus MIA+NS-R group; n = 6 per group. All data are presented as the mean ± SEM.

To further confirm the role of GlyT2 at the spinal dorsal horn in the regulation of acupoint sensitization, GlyT2-shRNA or Con-shRNA was injected to the left side of spinal cord dorsal horn at 21 days before MIA injection. Immunofluorescence confirmed that the GlyT2-shRNA (green fluorescence) localization was limited to cells on side of the spinal dorsal horn, and the protein level of GlyT2 on the left side decreased by almost 73% in the GlyT2-shRNA group compared to control group in normal rats (Figures 7(b) and 7(c)). However, the protein level of GlyT2 on the right side of the spinal dorsal horn in the GlyT2-shRNA group did not change in normal rats. Interestingly, GlyT2 was decreased at bilateral dorsal horns of spinal cord after unilateral (left) GlyT2-shRNA injection in KOA rats (Figure 7(d)). The extracellular glycine concentration was increased in the GlyT2-shRNA group after MIA injection (Figure 7(e)). The PWMTs of bilateral ST35 was also significantly elevated in the GlyT2-shRNA group compared to the Con-shRNA group (Figure 7(f)). For the nonsensitized acupoint GB37 and nonacupoint area, PWMT in the GlyT2-shRNA group was similar to that of the Con-shRNA group (Figures 7(g) and 7(h)). Consistently, GlyT2-shRNA reduced the number of mast cells and the MCDR in bilateral ST35 areas but not GB37 or nonacupoint areas (Figures 7(i) and 7(j)).

**Figure 8:**
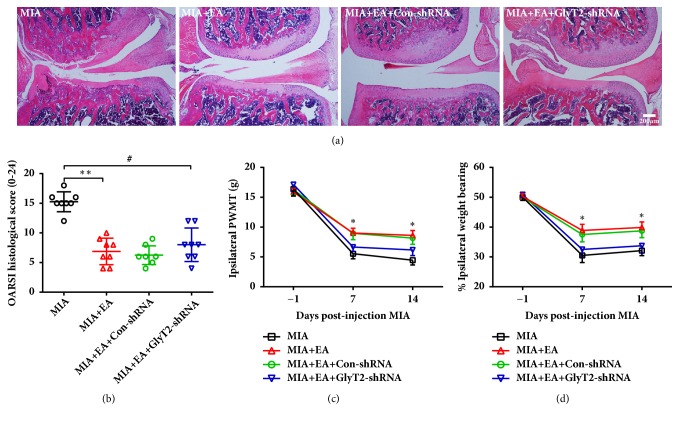
Downregulation of GlyT2 by GlyT2-shRNA partially reversed EA-induced analgesia in KOA rats. (a) Representative H&E staining for articular pathology in each group. The scale bar is 200 *μ*m. (b) OARSI scores are presented as the mean ± SD; Kruskal-Wallis test was used, *∗∗P* < 0.01 versus MIA group; ^#^*P* < 0.05 versus MIA group; n = 8 per group. ((c) and (d)) Paw withdrawal mechanical thresholds of the ipsilateral hind paw and weight-bearing deficits were assessed. Data are presented as the mean ± SEM; two-way repeated measures ANOVA followed by Bonferroni's post hoc test was used.

To further verify the acupoint function of sensitized ST35 during EA treatment for KOA, we applied EA treatment at bilateral ST35 acupoints to animals that accepted ipsilateral intraspinal injection of GlyT2-shRNA or control virus before KOA. The results showed that GlyT2-shRNA reversed the pain behavior alleviation induced by EA treatment (Figures 8(c) and 8(d)). However, the articular pathological lesions and OARSI score improvement induced by EA treatment were not blocked by GlyT2 knockdown (Figures 8(a) and 8(b)).

## 4. Discussion

**Figure 9:**
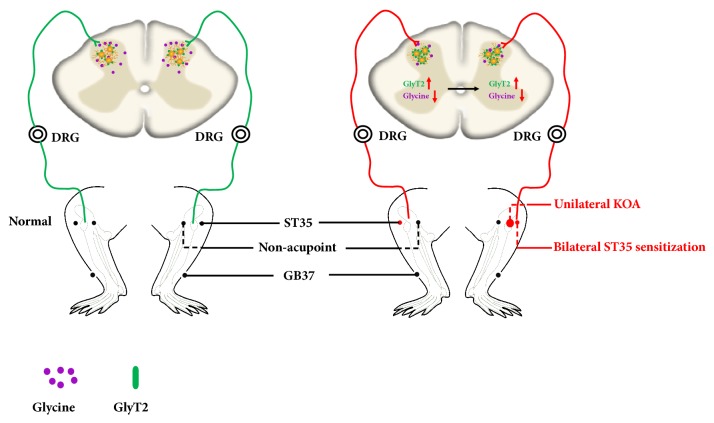
Illustration of mechanism underlying ipsilateral GlyT2 reduction-mediated bilateral ST35 sensitization in KOA rats. (a) Under physiological conditions, peripheral sensory input arises from the knee via the dorsal root ganglion and spinal cord dorsal horn up to the cerebral. Acupoints (ST35 and GB37) and nonacupoint area sensory input pathways are intact and exhibit normal reception of peripheral stimuli. (b) Under pathological conditions such as KOA, noxious stimuli induce firstly ipsilateral then contralateral GlyT2 expression elevation and reduced extracellular glycine concentration in both sides. These molecular alterations in the lumber spinal cord dorsal horn induce specific bilateral ST35, but not GB37 or nonacupoint area sensitization.

This study demonstrated that KOA induced bilateral ST35 sensitization and central sensitization while increasing GlyT2 expression and decreasing the extracellular glycine concentration in the bilateral dorsal horns of the spinal cord at L3-5. Either inhibiting GlyT2 function or reducing GlyT2 expression, especially on the ipsilateral of the spinal cord, was able to attenuate bilateral ST35 sensitization (Figure 9). These findings suggest that peripheral acupoint sensitization is modulated by the spinal cord and that elevated GlyT2 expression in the ipsilateral dorsal horn of the spinal cord may be a key mechanism for the induction of bilateral ST35 acupoints sensitization.

“Huang Di Nei Jing,” a well-known Traditional Chinese Medicine book in history, described “acupoint is where the pain lies.” This description is similar to the phenomenon of acupoint sensitization. However, it was not until recent years that the concept of “acupoint sensitization” was introduced [1]. In clinical practice, an important step in performing acupuncture is to find the sensitized acupoints. Sensitized acupoints can be used to induce better efficacy than nonsensitized acupoints. Sensitized acupoints may also have diagnostic therapeutic significance [28]. Acupoint ST35 is suggested to be sensitized acupoint in patients with KOA and acupuncture at ST35 has been demonstrated to ameliorate KOA-related pain and reduce joint destruction [29–32]. However, no well-accepted scientific indicators exist to test acupoint sensitization and the underlying mechanism is still unknown. Because the sensitized acupoint is much more sensitive to pressure stimuli than nonsensitized acupoint, in the current study, we first used the acupoint area mechanical threshold to judge whether acupoint sensitization or not. Results showed that bilateral ST35 but not GB37 or a nonacupoint occurred sensitization in KOA rats. Mast cells are often located in acupoint areas [33]. These mast cells secrete numerous mediators that activate sensory neurons, which in turn activate the mast cells by releasing neurotransmitters or neuropeptides [34]. The link between acupoint sensitization and mast cell degranulation has been observed empirically [15, 28, 35–37]. Our results showed that the total number of mast cells and the MCDR in bilateral ST35, but not in GB37 or nonacupoint areas, were significantly higher in KOA rats than in control animals. These tested points are all located in the L3-L5 somatosensory area, suggesting that sensitization induced by KOA is acupoint-specific.

The spinal cord is the first relay in the sensory pathways from the periphery to the brain. The CNS can be sensitized by noxious peripheral stimuli, after which it amplifies minor peripheral input into noxious stimuli [38]. Multiple lines of evidence support the concept that removal of glycine or GABA inhibition in the spinal cord leads to peripheral hyperalgesia [4]. Our previous report of a new mechanism of allodynia revealed that disinhibition of glycinergic neurons in the spinal cord is responsible for the hyperalgesia in a neuropathic pain model [39]. Similarly, Foster et al. found that peripheral hyperalgesia is accompanied by diminished inhibition of neurons in the dorsal horn [40]. Therefore, we presumed that the function of inhibitory neurons, especially glycinergic neurons, might be responsible for the acupoint-specific mechanical threshold reduction observed in KOA rats.

Glycine is a crucial inhibitory neurotransmitter that is released by both local interneurons and inhibitory descending fibers in the spinal cord. Glycine binds to strychnine-sensitive glycine receptors (GlyRs) and causes chloride ion influx into the postsynaptic cytoplasm. This influx results in hyperpolarization and raises the threshold for action potentials, thereby inhibiting the postsynaptic neuron. Aside from its inhibitory effects, glycine is also an essential coagonist of the N-methyl-D-aspartic acid (NMDA) class of glutamate receptor. Glycine also binds to NMDARs with 100-fold higher affinity than to GlyRs. Therefore, it was assumed that the glycine-binding sites of NMDARs are saturated under physiological conditions [41]. In this study, we found that the c-Fos increased but extracellular concentration of glycine decreased in the bilateral L3-5 spinal cord in KOA rats. However, intrathecal administration of glycine blocked peripheral acupoint sensitization phenomenon. Therefore, we believed that ST35 acupoint sensitization in KOA rats is mediated by central sensitization.

Extracellular glycine levels are controlled by the specific glycine transporters GlyT1 and GlyT2, which actively reuptake glycine from the synaptic cleft [42]. Both GlyT1 and GlyT2 subtypes belong to the sodium-dependent solute carrier family 6 (SLC6) of transporters, but they have different regional and cellular expression patterns in the CNS. GlyT1 is mainly expressed in astrocytes, with scarce expression on nonglycinergic neurons [43, 44]. GlyT2 is mostly present in the axon terminals of glycinergic neurons, where it is responsible for glycine uptake into the presynaptic cytosol [41, 45]. In the current study, we observed overexpression of GlyT2 but not GlyT1 in L3-L5 of the bilateral dorsal horns of spinal cord in KOA rats. Blockade of GlyT2 with ORG25543 or ipsilateral knockdown of GlyT2 expression in the spinal cord by shRNA resulted in increased glycine concentration in the extracellular space. Both manipulations also reversed the mechanical threshold reduction and mast cell degranulation elevation in bilateral ST35 but not in GB37 or the nonacupoint area. Therefore, we speculate that KOA decreases extracellular glycine concentration via increasing uptake by overexpressed spinal cord GlyT2, which in turn results in ST35 acupoint sensitization.

To investigate whether acupoint sensitization is involved in the therapeutic effect of EA against KOA, we used GlyT2-shRNA to desensitize ST35 in KOA rats. The results showed that EA could not ameliorate pain-related behaviors after ST35 desensitization, but it could still reduce joint lesion in KOA rats. This piece of evidence indicates that the spinal GlyT2-mediated ST35 sensitization is pivotal for EA's analgesia effect, but the joint lesion reduction by EA may involve local regulation as well as central regulation which does not require GlyT2 overexpression. A previous study used transcutaneous electrical nerve stimulation (TENS) at ST36 (Zusanli) and SP6 (Sanyinjiao) for rats with neuropathic pain and found that glycine concentration within the axon terminals in the spinal cord dorsal horn was significantly elevated compared to that of untreated or naive control rats [46]. This finding indicates that electric stimulation at certain acupoints may increase presynaptic glycine content in the spinal cord. However, whether the analgesic effect is generated by extra release of glycine into the synaptic cleft was not tested in their study. In the current study, knockdown of GlyT2 in the spinal cord disrupted the gateway of glycine reuptake and increased the extracellular glycine concentration, which in turn blocked peripheral ST35 sensitization. Knockdown of GlyT2 also blocked EA-induced pain relief in KOA rats, which further confirmed that spinal cord-mediated acupoint sensitization is important for EA-elicited pain relief. However, joint histopathological improvement induced by EA treatment was not reversed by intraspinal GlyT2 knockdown. These results suggest that the therapeutic effect of EA on joint pathology may be mediated by mechanisms other than the spinal cord glycinergic system. It has been reported that the endocannabinoid signaling pathway, beta-endorphin, and substance P are also involved in KOA-related pain relief by EA stimulation [47, 48].

In the current study, unilateral KOA, an inflammatory pain model, was used. Interestingly, unilateral KOA induced hyperalgesia in bilateral ST35 acupoints, but not GB37 or nonacupoint area. And this effect was mediated by bilateral excessive upregulation of GlyT2 expression. Up to now, we did not find any study that examined the expression of extracellular glycine or GlyT1/2 in KOA model. However, Villarejo-López et al. reported that the expression of GlyT2 was significantly increased in primary cultured neurons from the brainstem and spinal cord when treated by proinflammatory mediators or pain signaling molecules such as prostaglandin E2 (PGE2), substance P (SP), lipopolysaccharide (LPS), or ATP [49]. And in the study by Han et al., they found that the increased expression of GlyT2 was positively correlated with pain frequency in the sickle cell disease patients [50]. In KOA model, bilateral spinal cord changes caused by ipsilateral injury were reported previously. Lee et al. found that pERK1/2 was increased in the bilateral spinal dorsal horns in unilateral KOA [50]. And in unilateral KOA model, 2-4-fold increase in mRNA expression was found for endocannabinoid targeted receptors (Cnr1, Cnr2, and Trpv1), endocannabinoid degradation enzymes (Faah, Ptgs2, and Alox12), and TRPV1 sensitizing kinases (Mapk3, Mapk14, Prkcg, and Prkaca) in bilateral dorsal horns of spinal cord [51]. Additionally, GlyT2 has been investigated as an analgesic target in various pain conditions [5, 23, 52]. Another possible mechanism is that contralateral effects are mediated by overlapping central terminals of afferents or spinal interneurons [53]. Monosynaptic rabies virus tracing revealed that most of the afferent labeled spinal neurons were found in laminae II–V ipsilateral to the injection side, while a few scattered neurons were found on the contralateral side [54]. An ipsilaterally projecting interneuron has processes crossing the midline to the contralateral side [55], indicating a possible local transmedian projection for neuronal activity modulation. Taken together, bilateral spinal cord changes caused by unilateral injury have been observed in other pain models including nerve injury and inflammatory injury [56–63]. Despite the exact mechanism, GlyT2 as well as extracellular glycine that we have observed in KOA model was not reported elsewhere, we do believe that point is exactly one novelty of this study. Our evidence suggested that contralateral acupoint sensitization and GlyT2 changes as well as glycine variations were all derived from ipsilateral GlyT2 changes since knockdown of ipsilateral GlyT2 before MIA injection blocked all the manifestation in the contralateral side.

## 5. Conclusions

In summary, we first provide evidence showing that acupoint sensitization is an external manifestation of central sensitization and found that ST35 acupoint sensitization in KOA rats is induced by excessive glycine intake from the synaptic cleft by overexpressed GlyT2. The decreased level of glycine in the bilateral dorsal horns of the spinal cord is responsible for the sensitization of bilateral ST35 acupoints. And the analgesic effects of EA at sensitized acupoints require spinal GlyT2 expression. The exact mechanism of contralateral acupoint sensitization after KOA warrants further study.

## Data Availability

The original experimental data used to support the findings of this study were included within the article.
